# Saccadic Eye Movements in Depressed Elderly Patients

**DOI:** 10.1371/journal.pone.0105355

**Published:** 2014-08-14

**Authors:** Nicolas Carvalho, Nicolas Noiret, Pierre Vandel, Julie Monnin, Gilles Chopard, Eric Laurent

**Affiliations:** 1 Department of Clinical Psychiatry, University Hospital, Besançon, France; 2 E.A. 481, Laboratory of Neurosciences, University of Franche-Comté, Besançon, France; 3 CIC-IT 808 Inserm, Besançon University Hospital, Besançon, France; 4 E.A. 3188, Laboratory of Psychology, University of Franche-Comté, Besançon, France; 5 UMSR 3124/FED 4209 MSHE Ledoux, CNRS and University of Franche-Comté, Besançon, France; University of Ulm, Germany

## Abstract

The primary aim of this study was to characterize oculomotor performances in elderly depressed patients. The second aim was to investigate whether cognitive inhibition measured by the antisaccade task was associated with a psychomotor retardation or rather with a more specific cognitive-motor inhibition deficit. Twenty patients with a major depressive disorder and forty-seven healthy subjects performed two eye movement tasks. Saccadic reaction time and error rates were analyzed in the prosaccade task to obtain basic parameters of eye movements. Saccade latency, error rates and correction rates were evaluated in the antisaccade task to investigate inhibition capacities. Performances were impaired in patients, who exhibited a higher reaction time and error rates compared to controls. The higher time cost of inhibition suggested that the reaction time was not related to global psychomotor retardation alone. The higher time cost of inhibition could be explained by a specific alteration of inhibition processes evaluated by the antisaccade task. These changes were associated with the severity of depression. These findings provide a new perspective on cognitive inhibition in elderly depressed patients and could have important clinical implications for our understanding of critical behaviors involving deficits in inhibitory processes in the elderly.

## Introduction

Cognitive inhibition, a major component of executive functioning, has been found to be impaired in depressed patients [1], [2]. This function has been defined as a deletion of an automated response by the deliberate control of this response based on changes in background characteristics [3] or previously activated processes [4]. Cognitive inhibition would affect the characteristics of inputs and outputs of distracter elements to make the analysis and response processes for relevant elements available and more effective [5]. Houghton and Tipper [6] have shown that inhibition processes correspond to the cognitive function applied to the content that should be deleted.

An effective way to assess cognitive inhibition is based on oculomotor measurements particularly through the prosaccade and the antisaccade tasks [7]–[9]. Prosaccades are usually conceived as reflexive eye movements towards a peripherally appearing target. The prosaccade task has been used to evaluate basic characteristics of speed, latency and accuracy of saccadic eye movements. In contrast, the antisaccade task probes the ability to inhibit the automatic orientation of the eye in the direction of a peripherally appearing target in the vision fields [10]. Antisaccades are conceived as highly voluntary eye movements in the opposite direction to a peripherally appearing target. The antisaccade task is more reliable for assessing the inhibition function compared to many other tasks [11]. Antisaccade latency and error rates (ER) reflect critical cognitive abilities and can help us quantify an inhibition deficit [12].

A few reported studies have examined cognitive inhibition in depression. Sweeney *et al.* [13] showed that patients with depression - compared with healthy subjects - have greater difficulty in suppressing saccades towards a peripheral target in the antisaccade task. However, no difference in latency or speed was found. The study of Mahlberg *et al.* [14] showed that patients with depression had longer reaction times (RT) than controls in prosaccade tasks, suggesting that a psychomotor retardation affecting reflexive eye movement could characterize some depressive symptoms. The study conducted by De Lissnyder *et al.* [15] reported that dysphoric subjects had longer RT than controls in the antisaccade task. Globally, these results highlight the need to distinguish between basic reflexive components evaluated by prosaccade tasks and more elaborated executive processes involved in the inhibition required by the antisaccade task. A systematic distinction between these processes is critical to better characterize the kind of cognitive-motor processes that are altered in depression (i.e., global eye movement retardation *vs.* specific inhibitory disorder).

Moreover, all these studies only included young depressed patients. No information is available concerning oculomotor abilities in depressed elderly patients. Nevertheless, elderly depression differs from younger adult depression with a higher sadness, a psychomotor retardation, a difficulty to express their pain [16], a deficit in motor response and in response selection [17]. Depression in the elderly remains under-diagnosed [18] due to many co-morbidities such as somatic disorders [19], cerebrovascular pathologies [20] or Alzheimer’s disease [21].

The main objective of our research was to characterize oculomotor performances in elderly depressed patients. We hypothesized that depressed elderly patients would have a higher reaction time in both prosaccade and antisaccade tasks, because of global psychomotor retardation, which was previously reported in these patients. The second objective of this study was to determine whether higher antisaccade reaction time is only related to psychomotor retardation or if it is also due to a specific inhibitory problem. This is in accordance with the above mentioned alteration of response selection problems known in these patients.

## Methods

### 1. Participants

Elderly depressed patients were recruited from the psychiatric wards of Besançon University Hospital. Non-depressed controls were mainly recruited from the open University, which welcomes many older adults. Twenty depressed patients and forty-seven controls were included and matched for age (±5 years), sex, and education level. All patients had a major depressive disorder (MDD) according to the DSM-IV criteria [22], and a Montgomery-Asberg Depression Rating Scale (MADRS) [23] score higher than 25. Exclusion criteria for both groups were progressive psychiatric illness (e.g. schizophrenia, bipolar disorders), acute or chronic neurological pathologies (e.g. traumatic brain injury, brain tumors, stroke, and dementia) and presence of ophthalmic illnesses. Patients were all on stable medication at least for 4 weeks before inclusion screening. All patients were on antidepressants, 92% on anxiolytics, 80% benzodiazepines, 48% antipsychotics and 44% hypnotics. All participants gave their written informed consent to participate in the study. The research protocol was approved by the Committee for the Protection of Persons (CPP-Est-II), and was conducted in accordance with the principles laid down by the Declaration of Helsinki.

All participants underwent a complete neuropsychological battery of tests and eye movement tasks as described below. These assessments were performed within a maximum period of one month.

### 2. Neuropsychological assessment

The RAPID neuropsychological battery [24] was designed to ensure that no participant had cognitive impairments associated with early dementia. This battery included pencil and paper tasks assessing six cognitive domains: *(1) Global cognitive function*: Mini-Mental State Examination (MMSE) [25]; *(2) Attention/processing speed*: Trail Making Test Part A (TMT A) [26] and Crossing-Off Test (COT) [27]; *(3) Constructional praxis*: part of the Signoret’s battery of cognitive efficacy (BEC 96) [28]; *(4) Executive function*: Trail Making Test Part B (TMT B) [26] and Isaacs Set Test (IST) [29]; *(5) Verbal Memory*: Memory Impairment Screen (MIS) [30] and Free and Cued Selective Recall Test (FCSRT) [31], [32]; *(6) Language:* Picture naming task (DO 30) [24]. In addition to the RAPID battery, each participant completed the Stroop test [33] in order to measure inhibitory processing.

### 3. Eye movement tasks

All participants were seated in a quiet room. Eye movements were recorded using video-oculography techniques based on the corneal reflection of infra-red light. We used an ASL EYE-TRAC 6 system (ASL - Bedford, MA.) with a H6 optic camera mounted in a chin rest for stabilizing the head position. This system permits to capture data with good temporal (sampling at 120 Hz) and spatial resolution (accuracy of 0.1° of the visual angle). The device was calibrated for each participant at the beginning of the experimental sessions. The standard calibration of Eye-Track 6 User Interface Program (Version 1.62.1.0) with 3×3 calibration points was initially used. This was followed by an auto-calibration procedure, the 9 green points appearing one after another when the device automatically detected the subject’s eye. Each participant performed a prosaccade task and then an antisaccade task. These tasks were presented in two separate blocks, the prosaccade task before the antisaccade task. In the prosaccade task, participants were instructed to fix their gaze, as quickly and accurately as possible, on the red dot appearing in the periphery of the screen. In the antisaccade task, participants were instructed to fix their gaze, as quickly and accurately as possible, on the opposite side relative to the red dot appearing in periphery of the screen, [34]. Between the two tasks, an auto-calibration was then performed again.

**Figure 1:**
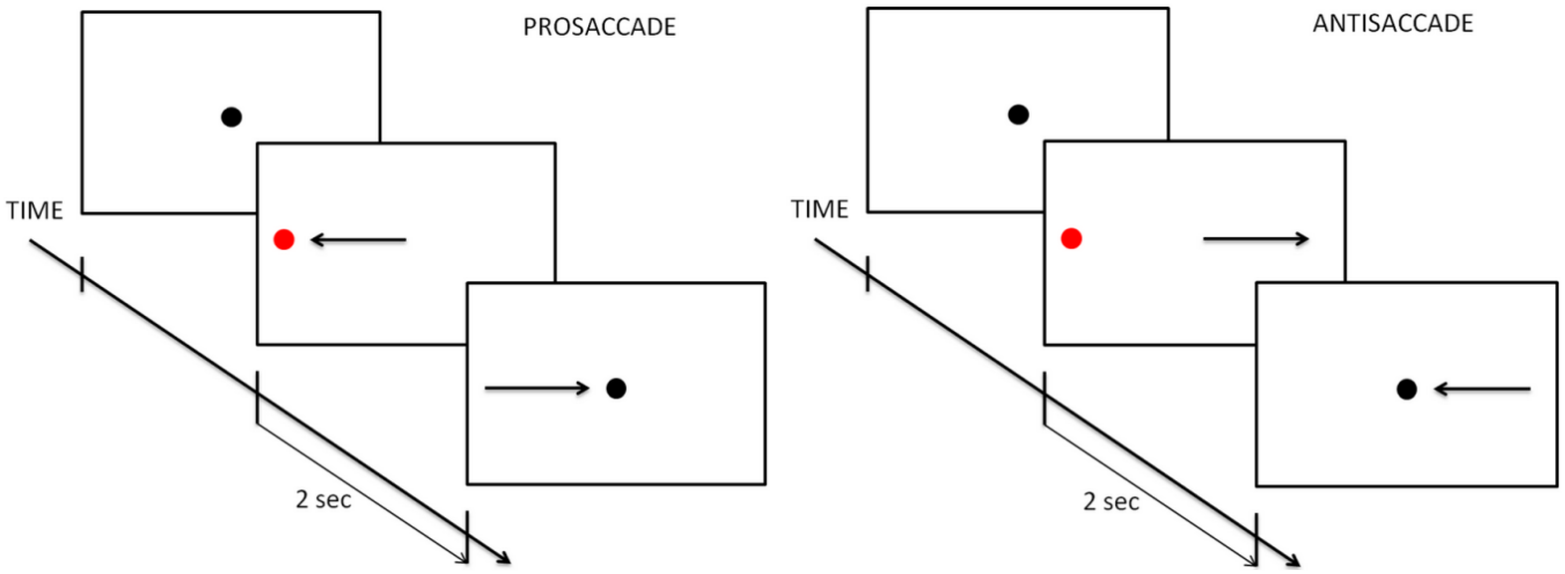
Eye movement tasks.

For both tasks, each trial began with a 2-s presentation of a central fixation dot. After this time, the peripheral red target appeared horizontally (H), on the right or left, or vertically (V), on the top or bottom, at different eccentricity levels (4°; 6°; 8°; 10°) in a randomized order (Figure 1) and for 2-s. The number of target eccentricity levels for the antisaccade task was limited in order to reduce its duration (4°; 8°). There were 4 trials in each condition of dot presentation. The central dot and the peripheral dot diameters each subtended a visual angle of 0.6°.

Pictures were presented on a 21-in. LCD screen (ASUS LCD Monitor VH 196, 32 bits color, 1024 by 768 pixels screen resolution, refresh rate of 70 Hz) at a distance of 70 cm from the participants. The images subtended a visual angle of 32.6°. Inquisit software (Millisecond Software, Version 3.0.3.2, Seattle, WA) was used to create the different tasks.

### 4. Eye Movement data analysis

Analyses were performed using the ASL-Result software. Fixations, total duration of fixation, and RT were computed on the basis of fixation detection criteria. Periods during which the eye did not vary from more than 1° of visual angle during at least 100 ms were considered as fixations. Accuracy zones around the red dot measured 1.8°.

#### Prosaccade

Recorded data were the RT (i.e., the time between the appearance of the peripheral target and the start of the correct subsequent saccade), the gain corresponding to the ratio of the saccade amplitude divided by the target step amplitude. A gain of <1 indicated that the saccade was hypometric and a gain of >1 indicated that the saccade was hypermetric.

#### Antisaccade

The RT, the ER (i.e., percentage of trials with initial incorrect saccade corresponding to a saccade toward the peripheral target), and the correction factor (CF) (i.e., sequence made of a first saccade toward the peripheral dot position and a second saccade in the opposite direction) were recorded.

When the saccade RT was lower than 80 ms (typically anticipatory saccade response) [35] or higher than 600 ms (typically delayed response), trials were excluded from data analysis [36].

### 5. Statistical analyses

A Shapiro-Wilk test was performed to assess the normality of data. The equality of variance was controlled by a Fisher-Snedecor test. Categorical variables were compared using the chi-square test or the Fisher’s exact test (if the sample size was less than 5). Mixed ANOVAs including the factor group (depressed patients *vs.* controls), direction (horizontal *vs.* vertical) and eccentricity (4°; 6°; 8°; 10°) were performed. Newman–Keuls post-hoc tests were applied when appropriate. The Greenhouse-Geisser correction was used when sphericity was not assumed.

Differences between both groups in the prosaccade and antisaccade tasks were evaluated using ANCOVAs including group (depressed, control) as a factor, and neuropsychological [All RAPID neuropsychological battery scores, MMSE, memory scores (free and total recall score of the FCSRT), information processing speed scores (Stroop M and C, TMT A, COT), executive functions scores (TMT B-A, IST, Stroop INT)] and psychiatric (MADRS) scores, as covariates.

The significance alpha level was fixed to 0.05. Effect sizes were measured by partial Eta squared (η^2^_p_), with small, medium and large effects defined as 0.01, 0.06 and 0.14 respectively [37]. All computations were performed using Stata Software release 10.1 (StataCorp, College Station, TX).

## Results

Demographic, psychiatric and neuropsychological data of the two groups are presented in table 1. There was no significant differences in age (depressed mean = 70.4, SD = 9.62; control mean = 66.72, SD = 5.48; *W*_67_ = 0.81, *p* = 0.41), gender (*χ* ^2^_(1)_ = 1.10, *p* = 0.29) or educational level (*χ* ^2^_(3)_ = 5.25, *p* = 0.21) between patients and controls. Scores on the MADRS were significantly higher in the patient group (MADRS: *W*_67_ = 6.36, *p*<0.001). Though patients with depression performed significantly worse than controls on most neuropsychological tests (Table 1), none of them had cognitive impairment sufficiently severe to constitute early dementia. As mentioned earlier, ANCOVAs were applied in order to control potential effects of neuropsychological performances on eye movements.

**Table 1:** Demographic and clinical data.

Characteristic	MDD(N = 20)	HC(N = 47)	p
Age	70.4±9.6	66.7±5.5	N.S.
Female	15(75)	29(61.7)	N.S.
Education (%)			N.S.
Low	25	21	N.S.
Medium	30	32	N.S.
High	45	47	N.S.
MADRS, (range 0–60)	31.7±5.6	2.3±2.3	<0.001
MIS Score, (range 0–8)	7.4±0.6	7.8±0.5	<0.001
IST Score,	31.3±5.3	43.3±5.8	<0.001
MMSE score, (range 0–30)	26.1±3.2	29.1±0.9	<0.001
FCSRT score, (range 0–48)			
Free recall	20.4±7.1	26.3±4.4	0.02
Total recall	43.4±6.8	45.7±2.5	N.S.
COT score,	145.6±48.4	213.7±42.1	<0.001
TMT score,			
Part A (range 0–150)	73.1±49.3	34.6±9.1	<0.001
Part B (range 0–300)	236.7±133.8	105.6±47.2	<0.001
BEC 96, (range 0–6)	5.6±1.1	6±0.2	0.03
DO 30, (range 0–30)	29.5±0.8	29.8±0.5	N.S.
Stroop Task score,			
Word	100±22.2	110.9±13.7	N.S.
Color	71.1±11.5	82.1±11.3	<0.001
Incompatible color-word	33.8±12.5	44±9	0.006
Interference	–8.3±9.5	–3.2±7.4	0.02

**Legend**: MDD, Major Depressive Disorder; HC, Healthy Controls; MADRS, Montgomery Asberg Depression Rating Scale; MIS, Memory Screen Impairment; IST, Isaacs Set Test; MMSE, Mini Mental State Examination; FCSRT, Free and Cued Selective Recall Test; COT, Crossing Off Test; TMT, Trail Making Test; BEC 96, Bec 96 figure Copy; DO 30, Picture naming task with 30 items; N.S., not significant.

Values given as *n* (%) or mean ± SD.

### 1. Differences in parameters of saccadic eye movements

#### 1.1. Prosaccade reaction time

Depressed patients had a significantly higher RT than controls [*F*(1,65) = 24.71, p<0.001, η^2^_p_ = 0.27, d = 1.3] (Table 2). There was no effect of direction [*F*(1,65) = 2.27, p = 0.14, d_s_<0.76], eccentricity [*F*(1,65) = 2.5, p = 0.11, d_s_<0.77] and no interaction between direction and eccentricity [*F*(1,65) = 0.91, p = 0.34, d_s_<0.43].

**Table 2:** Means and standard deviations for the saccadic reaction time, gain, error and correction rates.

Oculomotor parameter	MDD(N = 20)	HC(N = 47)	p	η^2^_p_
Prosaccades				
RT (ms)	291±59	231±28	<0.001	0.27
Gain	0.97±0.3	0.98±0.03	N.S.	<0.001
Antisaccades				
RT (ms)	405±116	284±54	<0.001	0.26
ER (%)	62±28.6	31±21.5	<0.001	0.27
CF (%)	83±17.6	83±24.9	N.S.	0.01

**Legend**: MDD, Major Depressive Disorder; HC, Healthy Controls; RT, Reaction time; ER, Error Rates; CF, Correction Factor; N.S., not significant.

Values given as mean ± SD.

#### 1.2. Prosaccade gain

The accuracy of prosaccade was not reduced in depressed group in comparison with the control group [*F*(1,65) = 0.02, p = 0.89, η^2^_p_<0.001, d = 0.04] (Table 2).

#### 1.3. Antisaccade reaction time

Depressed subjects had longer mean reaction times than controls [*F*(1,65) = 22.11, p<0.001, η^2^_p_ = 0.26, d = 1.33] (Table 2). There was a significant effect of direction [*F*(1,65) = 10.98, p<0.01, d_s_>0.28]. Horizontal RT was significantly higher for depressed patients (M = 420 ms, SD = 152) than healthy controls (M = 272 ms, SD = 52) (p<0.05; d = 1.30) but there was no difference in vertical direction (M_depressed_ = 326 ms, SD_depressed_ = 138; M_controls_ mean = 296 ms, SD_controls_ = 63; p = 0.22; d = 0.27). There was no effect of eccentricity [*F*(1,65) = 0.59, p = 0.44, d_s_<0.99] and no interaction between direction and eccentricity [*F*(1,65) = 0.05, p = 0.82, d_s_<0.04].

#### 1.4. Antisaccade error rate

Depressed patients had higher ER than controls [*F*(1,65) = 23.68, p<0.001, η^2^_p_ = 0.27, d = 1.22] (Table2). There was no significant effect of direction [*F*(1,65) = 0, p = 1, d_s_<1.15], eccentricity [*F*(1,65) = 0.001, p = 0.97, d_s_<0.99] and no interaction between direction and eccentricity [*F*(1,65) = 0.02, p = 0.87, d_s_<0.85].

#### 1.5. Antisaccade correction factor

Depressed patients and controls had a similar correction rate of antisaccade errors [*F*(1,65) = 0.99, p = 0.32, η^2^_p_ = 0.01, d<0.001] (Table 2).

### 2. Time cost of inhibition

In addition to direct measures of RT, the time cost of inhibition (antisaccade RT minus prosaccade RT) was calculated in order to control global eye movement retardation and provide a more reliable evaluation of the cognitive inhibition function (required in the antisaccade task). The time cost of inhibition was significantly higher in depressed patients (M = 114 ms, SD = 89) than in controls (M = 53 ms, SD = 42) [*F*(1,65) = 30.69; p<0.001].

### 3. Analysis of confounding variables (ANCOVA)

Due to the significant disparity in cognitive performances of the depressed patients and controls, we reanalyzed the eye movement performances explored in the two tasks using neuropsychological tests as predictor variables. All RAPID neuropsychological scores, MMSE, memory scores, information processing scores and executive function scores were not found to be significant predictors for eye movement performance differences (*F*_s_>0.01; *p*_s_>0.05).

With a MADRS score of 31.7 for the depressed patients and 2.3 for the healthy controls, our data were reanalyzed using MADRS as a predictor variable of eye movement differences. As reported above, a mixed ANOVA had indicated that depressed patients had significantly lower eye movements performances than controls except for prosaccade gain and antisaccade CF. After adjustment by the MADRS, the oculomotor performances between the two groups did not differ significantly except for prosaccade gain and antisaccade CF. These results confirmed an effect of MADRS score on eye movements (Table 3).

**Table 3:** Eye movement performances×MADRS interaction between the 2 groups.

		Group		
Covariable	Dependent variable	F_1,65_	p	η^2^_p_
MADRS	Pro RT	3.68	N.S.	0.05
	Pro gain	7.97	<0.01	0.11
	Anti RT	0.02	N.S.	<0.001
	Anti ER	0.5	N.S.	<0.01
	Anti CF	2.79	N.S.	<0.01
	Anti - Pro RT	0.96	N.S.	0.06

**Legend:** MADRS, Montgomery Asberg Depression Rating Scale; Pro, Prosaccade; Anti, Antisaccade; RT, Reaction time; ER, Error Rates; CF, Correction Factor; η^2^_p_: effect size; N.S., not significant.

## Discussion

In this study, oculomotor impairments were found in elderly depressed patients. In the prosaccade and antisaccade tasks, depressed patients had higher RT and ER than controls.

For both groups, the RT was longer in the antisaccade task than in the prosaccade task due to the incompatibility between the target position and the target-directed movement. This result was consistent with those found in other studies [38], [39]. Moreover, patients with depression had higher RT in both prosaccade and antisaccade task compared to controls.

We cannot deny the effect of global eye movement retardation, since we found significant differences in RT even in the prosaccade task. Many studies have shown altered cortical structures such as the cerebellum [40], [41], the dorsolateral prefrontal cortex [42], [43] and frontal eye fields [44] in depression that could play a role in the RT of eye saccade [45]–[47]. In our study, depressed patients also had higher ER in the antisaccade task. Alteration of motor adjustment could have played a role in the antisaccade task as well as in the prosaccade task [48].

However, we ensured that differences found in the antisaccade task were not only due to psychomotor retardation. First, we controlled neuropsychological performances. Second, we computed the time cost of inhibitory processes. Inhibitory processing is altered in patients with depression, which affects saccade latency. Inhibitory processing may be changed at the selection stage of the motor response [49] that is sent to the oculomotor system. Indeed, it has been suggested that an alteration of this function could be related to attentional biases observed in depression through its involvement in the selective attention process. It is also noteworthy that all differences that were reported concerning RT, gain and ER, in prosaccades, antisaccades, and in the differential measures specific to inhibition cost, were dependent on the MADRS scores, but globally not predicted by classic neuropsychological tests. Therefore, depression seems to be at the heart of the changes in eye movements, and not the result of more generic neuropsychological alterations. The relative independency of neuropsychological scores and eye movement performances in elderly depression constitutes eye tracking as a complementary tool for inhibitory capacity evaluation in depressive disorders.

However, our analyses suggest that psychomotor retardation was not the major cause of patients’ worse performances in the antisaccade task. The depressed elderly also exhibited reduced performances on the interference score of the Stroop test. These results suggest an inhibition deficit in depressed patients affecting interference performance. However, it may be questioned whether this is the same deficit that causes impairment on the antisaccade task. Indeed, the lack of correlation between the Stroop interference score and antisaccade minus prosaccade RT (*rho* = −0.34, *p* = 0.16), prosaccade gain (*rho* = 0.35, *p* = 0.15) or antisaccade error rates (*rho* = 0.32, *p* = 0.19) suggests that there could be an additional specific alteration of the inhibition mechanism at the level of the movement planning process [50]. The antisaccade task requires a motor response at the opposite of the target whereas the Stroop test requires an incongruent motor response [51]. In the Stroop test, inhibition corresponds to both the interference (the mechanism that prevents irrelevant information entered in memory) and inhibition (active suppression process). Several studies using different tasks to assess the inhibition showed that the correlations between the scores of these tasks were low or nonexistent [52], [53]. Several studies have reported a link between cognitive impairment and an inhibition deficit in depression [54]–[56] associated with pre-frontal cortex dysfunctions [57], [58], especially when executive functions were scrutinized [59], [60]. Other cognitive functions may also be linked to inhibition such as working memory [61], [62]. However, these results remain controversial [54]. Methodological differences regarding the sample size and the heterogeneity of inclusion criteria could explain these disparities. Generally, the involvement of executive functions and inhibition mechanisms would be most important but this not only depends on the different types of depression [63]–[65], but also on their severity [56] and the age at the first depressive episode [66], [67]. Additional efforts are needed to identify precise processes than underlie the changes in inhibitory capacities. For example, Crawford *et al.* [68] found that the RT in prosaccade and antisaccade tasks did not differ between patients with Alzheimer disease (AD) and control subjects. However, patients with AD had more ER in the antisaccade task. Moreover, these performances were correlated with the severity of cognitive impairment in AD patients [68], [69]. In our study, elderly depressed patients had an inhibition deficit characterized by an higher ER in the antisaccade task, although the difference between depressed patients and controls in prosaccade and antisaccade RT seems to be related both to the effect of inhibition impairment and psychomotor retardation. These results were correlated with severity of the depression but not with cognitive impairment. Therefore, the current results may open new windows on specific moderation of the prefrontal cortex by affective circuits, possibly through amygdala-prefrontal cortex connections [70], even when stimuli are not emotional in nature. Current research also provides us with information about the specificity of motor inhibition impairment in elderly depressive patients, since this impairment is relatively independent from more cognitive-verbal processes measured by classic neuropsychological tests (e.g., Stroop test).

Inhibition deficit has been shown to be related to suicide risk in elderly depressed patients [71]. Cognitive inhibition is involved in decision-making and could help the depressed patient in preventing late-life suicide [72]. Cognitive inhibition could also reduce the intrusion of suicidal ideation and rumination [71]. Alexopoulos [73] suggested that the presence of cognitive inhibition alteration could be predictive of a lack of therapeutic response to antidepressants in the depressed elderly. Moreover, Malsert *et al.* [74] reported that the RT and ER performances in antisaccade task were associated with the severity of depression and could predict response to transcranial magnetic stimulation over the dorsolateral prefrontal cortex. Our results suggest that the oculomotor performances evaluated by the RT and the ER could be useful measurements of cognitive inhibition in elderly depressed patients. These performances are *complementary* to classical neuropsychological measurements since they seem to be at least partially independent. Further research would be required in order to confirm the interest of using eye movement measurements to predict treatment response or suicidal risks in elderly patients.

A possible limitation of this study, which is common to many experiments in the field, is related to the effect of drugs. Various studies have shown that drugs can affect eye movements [75]. Fafrowicz *et al.* [76] have highlighted an increase in saccadic RT in healthy volunteers treated by anxiolytic drugs. Green *et al.* [77] have also demonstrated an increase in antisaccade errors in schizophrenic patients treated by benzodiazepine. However, other series did not show any treatment effects on eye movements [78] or latency and ER for antisaccade in schizophrenic and depressed patients [79]. To reduce intervening effects, we ensured that all patients were in a stable phase of their disease and were not showing any clinical signs of drug side effects.

In conclusion, the results of this study have offered a new insight into the cognitive-motor inhibition impairment of elderly depressed patients. We used two simple eye movement tasks and additional data analysis focusing on the cost of inhibitory processes. Elderly depression was never previously studied based on this methodology. From a theoretical point of view, this questions the validity of a monolithic approach to cognitive inhibition.More specific conceptual categories are needed in order to account for the multiplicity of inhibitory processes and behaviors. From a clinical point of view, implications may include a more precise evaluation of inhibitory capacities in patients. Complementary research is needed in order to measure the predictive power of the manipulated variables on ecologically-relevant behaviors (i.e., suicidal risk, therapeutic response to treatment).
